# Genetic resilience or resistance in poultry against avian influenza virus: mirage or reality?

**DOI:** 10.1128/jvi.00820-25

**Published:** 2025-06-30

**Authors:** Paula R. Chen, Stephen N. White, Lianna R. Walker, Darrell R. Kapczynski, David L. Suarez

**Affiliations:** 1 Plant Genetics Research Unit, Agricultural Research Service, USDA 57836 , Columbia, Missouri, USA; 2 Poultry Microbiological Safety and Processing Research Unit, US National Poultry Research Center, Agricultural Research Service, USDA 17123, Athens, Georgia, USA; 3 Exotic and Emerging Avian Viral Disease Research Unit, US National Poultry Research Center, Agricultural Research Service, USDA 17123, Athens, Georgia, USA; Universiteit Gent, Merelbeke, Belgium

**Keywords:** interferon, MHC haplotype, resistance, resilience, chicken, influenza

## Abstract

The unprecedented global spread of the highly pathogenic avian influenza (HPAI) virus in wild birds, poultry, and mammalian species has challenged our control efforts. Alternative approaches to limit avian influenza viruses (AIV) include the development of resilient or resistant chickens. Genetically resilient birds may become infected but can overcome disease, whereas resistant birds prevent virus attachment or entry and do not become infected. The most intensively studied host gene is myxovirus-resistance (*Mx*), which is expressed via the interferon pathway. Both sensitive and resistant chicken *Mx* genotypes have been described, but this only provides limited resilience. Acidic nuclear phosphoprotein 32 family member A (*ANP32A*) has been demonstrated as a host cofactor for AIV replication via interaction with the polymerase. Small nucleotide changes within this gene have demonstrated some promise for the establishment of disease resilience. Certain MHC-defined genetic chicken lines have demonstrated increased resilience with higher innate immune responses, but HPAI-infected birds still have high morbidity and mortality. Alternatively, gene-edited or -transgenic chickens have had some success in increasing resilience. This strategy allows flexibility to include foreign genes, modification of existing genes, or combined approaches to block critical steps in the viral life cycle. Some candidate genes include solute carrier 35A1 (*SLC35A1*), retinoic acid-inducible gene I (*RIG-I*), and toll-like receptors 3 and 7 (*TLR3/7*), but animal testing needs to be conducted. Furthermore, existing hurdles for technology transfer to commercial application from either naturally occurring resistance genes or foreign genes remain high and will require acceptance by both the poultry industry and consumers.

## INFLUENZA VIRUS OVERVIEW

The natural reservoirs for type A influenza viruses are primarily wild waterfowl and gulls, but the virus can infect a broad host range, including mammals, due to the use of ubiquitous sialic acid as a host receptor (1). If the virus can transmit to new hosts and replicate, it can rapidly mutate to increase adaptation and transmissibility within that species and eventually become endemic in the new host population. In migratory waterfowl, the virus is predominantly an enteric virus, which is rarely recognized to cause disease but can be transmitted to chickens and turkeys, resulting in a range of clinical diseases depending on the viral strain (2).

Two pathotypes of avian influenza virus (AIV) are recognized based on the ability of the virus to cause disease in experimentally infected chickens: low pathogenic avian influenza (LPAI) and highly pathogenic avian influenza (HPAI). The LPAI viruses are primarily restricted to replication on mucosal surfaces, and although they may be highly infectious, they rarely cause severe disease in otherwise healthy birds. However, the HPAI viruses can replicate both mucosally and systemically, which results in more severe disease, often accompanied by high mortality rates (1). Despite the identification of 17 different hemagglutinin subtypes for AIV, only certain H5 or H7 strains are classified as HPAI viruses. The primary genetic difference between LPAI and HPAI viruses is the sequence at the HA cleavage site, where the substitutions of basic amino acids or insertions of amino acids enhance the ability of host proteases to cleave the HA protein into the HA1 and HA2 subunits, which is required for the virus to become infectious (3). The conversion from LPAI to HPAI virus is thought to primarily occur in chickens and turkeys after the virus has been transmitted from wild birds. Typically, only LPAI viruses circulate in wild bird populations, but a recent exception has disrupted this paradigm, whereby HPAI viruses of the H5 goose/Guangdong lineage have become endemic in wild birds, poultry, and multiple mammal species throughout the world (1). This lineage of the HPAI virus is defined exclusively by the hemagglutinin gene because of multiple reassortments of all the other gene segments. The goose/Guangdong lineage of viruses has remained highly virulent in chickens and turkeys and has infected or caused the depopulation of more than 170 million birds over the last 3 years in the United States alone. Additionally, there is heightened concern for potential zoonotic transmission of AIV, which is well-documented for H9N2, H7N9, and the goose/Guangdong lineage of viruses (4–6). However, the virulence and the ability to infect wild birds have changed considerably in the last 30 years, with some variants having an increased host range and/or increased clinical disease (7).

With recurrent and sustained HPAI virus infections of poultry worldwide, alternative control strategies are being considered aside from biosecurity, rapid detection, and depopulation, which can be effective but costly, and vaccination, which has been used with mixed success. An ideal solution would be to develop chickens and turkeys that are resistant to infection and maintain the current high level of productivity in modern commercial lines. However, despite considerable amounts of research on this topic, no poultry have been identified that are fully resistant to AIV infection. Although a handful of genes have been discovered that may reduce viral replication and/or control disease severity and mortality, the goal of a completely resistant chicken to AIV infection remains elusive. This review attempts to provide a better understanding of currently known genes and MHC haplotypes that may be important in developing an AIV-resilient or -resistant chicken through either classical genetic selection, gene editing, or transgenic technologies.

## RESILIENCE VERSUS RESISTANCE

When evaluating birds with enhanced heritable genetic capabilities against AIV, two main goals can be described, disease resilience and disease resistance (8). Disease resilience is the demonstration of reduced disease in the animal and enhanced ability to recover from infection. In poultry production, disease resilience can help maintain bird performance, leading to improved animal health with sustained production cycles. Although disease resilience does not prevent virus infection, the heritable genetic background of the bird contains characteristics to control, overcome, and resolve the disease. Within this context, the pathotype of the infecting virus involved, LPAI or HPAI, is an important consideration. Because LPAI virus infection in poultry (predominantly H1, H2, H3, H5, H6, H7, or H9 subtypes) generally does not result in high mortality, resilience from disease would provide increased protection from production losses and flock maintenance (9). In contrast, disease resilience from the HPAI virus (H5 or H7) is defined by the survivability of the flock during the infection to reduce mortality, improve animal welfare, and reduce transmission potential to uninfected birds or mammals. During HPAI virus infection, mortality generally occurs within 2–7 days (3). During this time, the innate immune response is activated, which includes the production of cytokines and interferons. Because the adaptive immune response, including both humoral and cellular immunities, is only becoming functional during this time, susceptibility to systemic virus replication in multiple organs and systems can lead to rapid mortality. Thus, it is widely accepted that the innate immune response is not sufficient to control the disease, and in the absence of preexisting neutralizing antibodies, high mortality is expected if commercial birds become infected (10). Although disease resilience from the HPAI virus does not stop infection, genetic changes that reduce virus replication, control inflammatory responses, or target viral pathways have been described and will be further discussed.

Disease resistance can be defined as the inability of an animal to be infected and, therefore, eliminate disease and transmission. Examples where resistance has been documented include diseases such as porcine respiratory and reproductive syndrome (PRRS) virus, bovine viral diarrhea virus, and avian leukosis virus, whereby resistance was conferred by deleting the viral receptor from the host, which prevented viral attachment and infection (8). In poultry production, disease resistance would result in animals that are non-permissive to direct or indirect infection from AIV (both LPAI and HPAI), leading to a disease-free status and preservation of overall animal health. However, AIV uses sialic acid as the host receptor, which is a post-genetic modification of most host proteins; therefore, a focus on the complete deletion of sialic acid residues is unlikely to produce viable animals.

## GENETIC MECHANISM OVERVIEW

**Fig 1:**
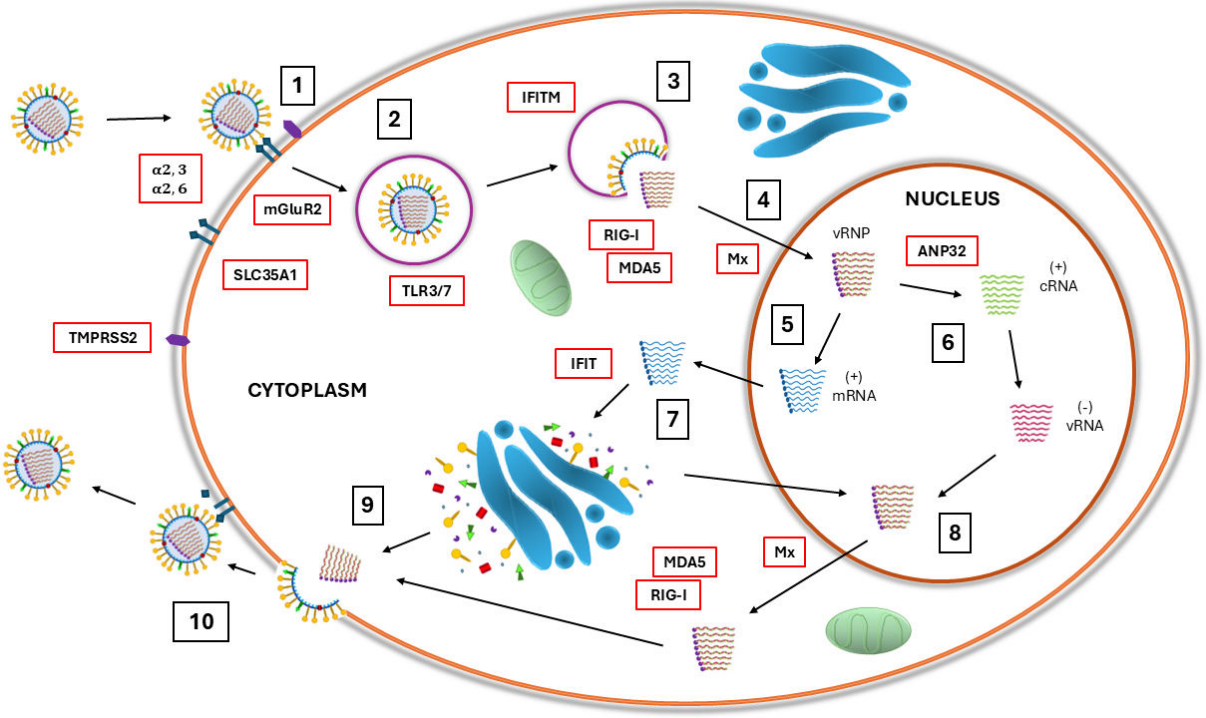
Host factor (red boxes) involvement across stages of avian influenza virus infection: (1) viral attachment to the host cell surface achieved by the binding of viral hemagglutinin to the host sialic acid receptors, (2) internalization of bound virions using clathrin-mediated endocytosis via specific receptors, (3) endosomal acidification triggers conformational change of cleaved hemagglutinin to facilitate membrane fusion and viral. uncoating, (4) viral ribonucleoprotein (vRNP) complexes released into the cytoplasm are translocated to the nucleus, (5) transcription of viral mRNAs, (6) replication of the viral genome, (7) translation of viral mRNAs to produce viral proteins, (8) encapsidation of the replicated viral genome segments forming vRNP complexes, (9) assembly of viral proteins and packaging of vRNP, and (10) budding of new virions and release from host cell surface following cleavage of sialic acid receptors by viral neuraminidase.

Viruses are obligate intracellular parasites that rely on host genes to support virus replication; therefore, one approach to control the virus is to identify critical host genes to allow targeted interventions. The following sections provide an overview of multiple mechanistic steps where host genetics may play a role in developing some form of genetic resistance or resilience, several of which also contribute to some level of species specificity for influenza A viruses (11). Multiple host mechanisms exist to detect viral pathogens, and blocking viral entry is an appealing route toward creating genetically resistant animals. Additional host mechanisms provide essential components co-opted for viral replication. Furthermore, host responses to viral infection include both cellular responses, such as interferon responses to broadly limit viral replication, and also highly specific adaptive immune responses. An overview of these mechanisms is provided in Fig. 1, and a review of relevant research is presented based on the timing of the viral replication cycle.

## VIRAL ENTRY

### Solute carrier 35 member A1 (SLC35A1)

The surface hemagglutinin protein on AIV binds to sialic acid residues on the host cell as the first step in viral entry. All sialic acids are nine-carbon molecules, and importantly, the second carbon atom of the sialic acid can be linked to the third carbon of the secondary terminal galactose via an α-2,3 sialyltransferase or to the sixth carbon via an α-2,6 sialyltransferase. The distribution and abundance of the different types of sialic acid residues in tissues are critical for determining the host range and can limit the cross-species transmission of particular strains (12). In most cases, AIV preferentially binds α-2,3 sialic acid residues, whereas human influenza strains preferentially bind α-2,6 sialic acids (13). However, there are many exceptions to this simple paradigm, including some hosts, like pigs, expressing both α-2,3 and α-2,6 sialic acids, differential expression of sialic acid based on tissue type, and the complex structure of sialic acid that allows some viruses to attach to both types of sialic acids (14). The cytidine 5′-monophosphate (CMP)-sialic acid transporter encoded by the solute carrier 35 member A1 (*SLC35A1*) gene is localized to the Golgi apparatus and is responsible for a large portion of sialic acid additions to numerous proteins. Several screens for host genes have identified this transporter as a facilitator of influenza A virus infection in humans, pigs, and chickens (15–19). However, natural mutations in *SLC35A1* were linked to the development of macrothrombocytopenia and neutropenia in humans (20), and endothelial-specific knockout of *SLC35A1* in mice resulted in excessive hepatic lipid accumulation and increased mortality (21). Thus, targeting this gene as a strategy to confer AIV resistance or resilience in chickens may prove challenging. It is possible that tissue-specific knockout of *SLC35A1*, such as in the respiratory tract*,* might become broadly useful in poultry, especially against LPAI viruses.

### Clathrin-mediated endocytosis

After attachment of the virus to host sialic acid, the virus is internalized primarily by clathrin-mediated endocytosis (22). In A549 cells, RNA interference (RNAi) screens were used to identify metabotropic glutamate receptor subtype 2 (mGluR2) and potassium calcium-activated channel subfamily M alpha 1 (KCa1.1) as key factors for clathrin-mediated endocytosis of AIVs (23). Specifically, mGluR2, which is encoded by the *GRM2* gene, binds to viral hemagglutinin and facilitates clathrin-mediated endocytosis. Meanwhile, KCa1.1, which is encoded by the *KCNMA1* gene, regulates the polymerization of F-actin to allow clathrin-mediated endocytosis to proceed. An additional factor, claudin-11, has been identified that promotes the dissociation of KCa1.1 from mGluR2 to allow for the maturation of the clathrin-coated pits (24). Knockout of *Grm2*, thus mGluR2, in mice decreased the replication of AIVs in the respiratory tract and improved survival after infection (23). Similar studies have not been performed in chickens, but chickens also have the *GRM2* gene (bGalGal1.mat.broiler.GRCg7b: NC_052543.1), which may present an additional target to increase genetic resilience.

## ACTIVATION BY HEMAGGLUTININ CLEAVAGE

### Transmembrane protease, serine 2 (TMPRSS2)

A key step for influenza virus entry is cleavage of the hemagglutinin protein by host proteases that free the fusion domain to allow membrane fusion (25). Host proteases, specifically trypsin-like proteases, expressed by epithelial cells in intestinal or respiratory tissues can perform this cleavage for LPAI viruses (25), and the transmembrane protease serine 2 (TMPRSS2) has been identified as the likely protease in avian and mammalian cell lines (26, 27). In a *TMPRSS2* knockout pig challenge model with two swine influenza A viruses (H1N1 CA04 and H3N2 TX98), the piglets had reduced nasal shedding (28–33). Although more data have been generated for mammalian models, several potential proteases have been identified in chickens (34). Future work will determine whether TMPRSS2 plays an important role in hemagglutinin cleavage in poultry, and whether TMPRSS2 or other proteases might influence genetic resistance to LPAI viruses. However, the multibasic cleavage site of HPAI viruses is cleaved by furin or other ubiquitous proteases that are present in all cell types (25); thus, TMPRSS2 is not involved in the activation of these strains, and ablation would not provide resistance in poultry.

## INNATE IMMUNITY/RECOGNITION

Pattern recognition receptors (PRRs), including toll-like receptors (TLRs) and retinoic acid-inducible gene 1 (RIG-I)-like receptors, that recognize diverse pathogen-associated molecular patterns (PAMPs) are key factors of innate immunity (35). Although some mammalian PRRs that detect viral nucleic acids are absent across avian species, such as RIG-I and TLR8, others differ from species to species, underscoring observed differences in pathogenesis following AIV infection (36, 37).

### Toll-like receptors 3 and 7 (TLR3 and TLR7)

Toll-like receptors (TLRs) are type I transmembrane proteins expressed on the cell surface or within cytoplasmic endosomes/lysosomes (35). In chickens, three endosomal TLRs detect intracytoplasmic viral nucleic acids in the form of single-stranded (ss) RNA (TLR7), double-stranded (ds) RNA (TLR3), and CpG DNA (TLR21) (38). Although dsRNA intermediates do not reach detectable levels during influenza viral replication (39, 40), TLR3-mediated immune responses have been widely demonstrated for influenza (41–43), as well as other ssRNA viruses, including SARS-CoV-2 (44) and porcine reproductive and respiratory syndrome (PRRS) virus (45). Studies have shown that TLR3 is also able to recognize highly structured ssRNA segments containing stem structures with bulges and internal loops (45, 46). Upon activation, TLR signaling is initiated by toll/interleukin-1 receptor (TIR)-domain-containing adaptor molecules, leading to the expression of type I interferons and pro-inflammatory cytokines (35). Specifically, TLR7 recruits myeloid differentiation primary response 88 (MYD88) to activate the transcription factors, interferon regulatory factor 7 (IRF7), and nuclear factor kappa B (NF-kB). In contrast, TLR3 signals through a MYD88-independent pathway by recruiting TIR-domain-containing adaptor-inducing interferon B (TRIF), leading to the activation of interferon regulatory factor 3 (IRF3) and NF-kB. Although birds appear to lack IRF3, avian IRF7 has a similar structure to mammalian IRF3 and may function analogously downstream of TLR3 signaling (47).

*In vitro* infection of chicken peripheral blood monocyte-derived dendritic cells demonstrated increased mRNA expression of all three nucleic acid sensing TLRs (3, 7, and 21) 24 h post-infection with H5N1 HPAI viruses, but not H9N2 LPAI viruses (48). However, treatment of chickens with ligands for TLR3 and TLR21 prior to exposure to H9N2 LPAI virus resulted in decreased fecal shedding (49), highlighting TLR3 and others as potential targets for limiting both HPAI virus and LPAI virus transmission. Although TLR3 and TLR7 are present in both chickens and ducks, distinct expression patterns were observed in response to AIV infection that were tissue- and strain-specific (47). The genetic factors underlying these differences remain unclear, as well as their ultimate impact on AIV resilience.

### Retinoic acid-inducible gene I (RIG-I) and melanoma differentiation-associated protein 5 (MDA5)

After the AIV genome is released within a cell, the viral RNA can be detected by different cytoplasmic sensors, including RIG-I and melanoma differentiation-associated protein 5 (MDA5). Both are classified as ATP-dependent DExD/H box RNA helicases, and RIG-I recognizes short single-stranded (ss) or double-stranded (ds) RNA (<500 bp), whereas MDA5 recognizes long dsRNA (>500 bp). Following activation, the caspase activation and recruitment domains (CARDs) of RIG-I or MDA5 bind to mitochondrial antiviral signaling protein (MAVS) to initiate expression of target interferon-stimulated genes as part of the innate immune response (50). Both genes are evolutionarily conserved and are present in avian species that are natural reservoirs for AIV, such as waterfowl, and have been hypothesized to be one of the reasons ducks are more resilient to clinical disease (51). Chickens and other galliforms (turkeys, quail, and pheasants) have a functional MDA5; however, this group has been reported to have lost the *RIG-I* gene approximately 45–65 million years ago, as well as the ring finger protein 135 (*RNF135*) gene, which encodes a cofactor for RIG-I (51). Importantly, the loss of *RIG-I* and its cofactor may partially explain the enhanced virulence of HPAI in chickens. Several *in vitro* studies have been performed to confirm the roles of MDA5 and RIG-I in AIV infection. When chicken DF-1 fibroblast cells expressing duck *RIG-I* were challenged with an HPAI virus, increased expression of *IFN-β* and other interferon-stimulated genes was observed along with decreased viral replication (52). Moreover, *MDA5* knockout DF-1 cells challenged with AIV demonstrated a dramatically reduced interferon response (53). When both *MDA5* and *TLR3* were knocked out, the cells did not mount a detectable interferon response. It remains to be determined whether transgenic insertion of a *RIG-I* sequence into the chicken genome will confer any degree of protection against avian influenza or be detrimental to the host.

## INTERFERON RESPONSE

Recognition of viral PAMPs by PRRs leads to cellular secretion of type I interferons (IFN-α and IFN-β), which are powerful inducers of an antiviral state as evidenced by the extreme susceptibility of type I IFN receptor knockout mice to numerous viruses (54). Type I IFNs mediate their protective effect by inducing or enhancing the expression of other genes, called interferon-stimulated genes (ISGs). Examples of well-known antiviral proteins encoded by ISGs are myxovirus resistance (Mx), interferon-induced protein with tetratricopeptide repeats (IFIT), and interferon-induced transmembrane (IFITM) gene families; however, several others have also been implicated in influenza A infection (55–57). An influenza virus gene encodes for the non-structural protein 1, which has the primary purpose of suppressing the interferon response, highlighting the importance of the interferon antiviral role (10, 58).

### Myxovirus resistance (Mx)

Myxovirus resistance (Mx) proteins are large GTPases that are produced in response to type I IFNs (59) and are key mediators of the innate antiviral response that directly interact with viral proteins to block replication and/or transcription of several DNA and RNA viruses (60–62). A functional GTP-binding motif and GTPase activity are necessary for the antiviral activity of Mx proteins (63). Mx proteins self-assemble into oligomeric rings, which are critical for GTPase activity, protein stability, and antiviral activity (64, 65). Mouse Mx1 (Mu Mx1) localizes in the nucleus and is a critical determinant of resistance to influenza A virus *in vivo* and *in vitro* (60, 63, 66). In challenge studies, *Mx1*-expressing mice are protected from a lethal dose of HPAI virus, whereas *Mx1*-negative mice succumb to infection (67, 68). However, a key difference in mouse and chicken HPAI infection studies is the longer mean death time in mice, which provides more opportunities for innate and adaptive immune responses to operate. Nuclear Mu Mx1 inhibits influenza A viral polymerase activity during primary transcription via interactions with the viral nucleoprotein (NP) (69–71). The human homolog with antiviral activity, MxA, is located in the cytoplasm and has a broader range of antiviral activity, including influenza A virus, vesicular stomatitis virus (VSV), measles virus, and hepatitis B virus (60, 62, 72).

The role of chicken Mx as an effective antiviral protein is highly contested. Chickens have a single *Mx* gene (Ck *Mx*) that is induced by type I IFNs (73). It was originally reported that chicken Mx lacked any antiviral activity *in vitro* (74). The Ck Mx protein was found to be highly polymorphic, with some breeds demonstrating some antiviral activity against influenza A virus and VSV (75, 76). Specifically, the presence of serine at residue 631 (S631) has been associated with the lack of Ck Mx antiviral activity, whereas an asparagine at this position (N631) conferred antiviral activity (75, 76). The susceptible allele (S631) was found in ancestral breeds but appears to be more prevalent in modern commercial broilers compared with egg-laying lines (77). Several reports have since negated that the S631N substitution conferred antiviral activity *in vitro* and *in vivo* (73, 78–80). One group reported that Ck Mx containing the N631 allele did not inhibit AIV replication in chicken embryo fibroblasts (CEFs) and that the cytoplasmic location of Ck Mx was not the reason for the lack of antiviral activity (78, 79). Another study reported that Ck Mx lacks GTPase activity, and therefore, has no antiviral activity against AIV (73). An *in vivo* study reported that chickens with N631 had no significant differences in pathology after the challenge with LPAI virus (81). However, another *in vivo* study reported that chickens homozygous for N631 had increased resilience to HPAI virus, but ultimately, all birds at higher challenge doses succumbed to disease (80). Although *in vitro* and *in vivo* experimental results vary on the effectiveness of Ck Mx as an antiviral effector, the fact remains that chickens are highly susceptible to certain lineages of AIV.

### Interferon-induced protein with tetratricopeptide repeats (IFIT) and interferon-induced transmembrane (IFITM)

Interferon-induced protein with tetratricopeptide repeats (IFIT) proteins are cytoplasmic enzymatically inactive proteins containing multiple repeats of the tetratricopeptide repeat (TPR) structural motif, which mediate protein-protein interactions and the formation of large protein complexes (57). A majority of cell types do not express IFIT under basal conditions and require interferon stimulation to induce gene expression (55, 57). Four members have been identified in humans (IFIT1, 2, 3, and 5), with evolutionarily conserved orthologs identified in other mammals, birds, fish, and amphibians (57). Homo- and hetero-dimerization of IFIT proteins have been shown to exhibit both non-specific and virus-specific antiviral activity by inhibiting translation initiation, binding uncapped or partially capped viral RNA, and sequestering cytoplasmic viral RNA/proteins (55). Recent research indicates that IFIT2 may promote antiviral responses by enhancing, rather than inhibiting, the translation of cellular mRNAs, which are enriched for ISG transcripts following viral infections (82). However, IFIT2 was also shown in this study to enhance the translational efficiency of influenza A viral mRNAs, resulting in a pro-viral rather than antiviral function of this protein during influenza infection (82). Ducks and chickens have a single IFIT gene (avIFIT), which shares the greatest homology with human IFIT5 and similarly inhibits viral replication by interacting with 5’-ppp viral RNA (47, 83). Chicken avIFIT was found to be upregulated in the intestinal and lung epithelial cells following infection with either LPAI or HPAI virus, respectively (84, 85). Overexpression of duck avIFIT in DF-1 cells lacking endogenous chicken avIFIT expression significantly reduced the replication of both LPAI and HPAI H5N1 viruses (86). However, cell growth was greatly impacted as a result of duck avIFIT expression in both AIV- and mock-infected cells (86).

Contrary to IFIT, the interferon-induced transmembrane (IFITM) family members are transmembrane proteins constitutively expressed in many cell types (55). In humans, IFITM1, 2, and 3 are highly upregulated in response to IFN signaling and exhibit antiviral activity (87). Restriction of infection by IFITM has been demonstrated for multiple enveloped viruses in which fusion with host cell membranes occurs in late endosomes or lysosomes (56, 88). Specifically, IFITM3 localizes to late endosomes and restricts influenza A virus replication by preventing the release of vRNP into the cytosol (89). Orthologs for the three antiviral IFITM genes have been identified in both ducks and chickens (37, 47). The ability of chicken and duck IFITM3 to restrict AIV infection has been demonstrated *in vitro*, supporting a similar function of IFITM in birds as observed in mammals (90, 91). However, dramatic differences in IFITM expression have been reported between species following AIV infection (37). Specifically, IFITM1, 2, and 3 were substantially upregulated in the lung and ileum of ducks following H5N1 HPAI infection, whereas chickens exhibited minimal IFITM upregulation (37). Whether due to differences in PRR expression and/or IFITM genetic variation, the limited response observed in chickens may represent a key factor conferring increased susceptibility to AIV infection.

## VIRAL REPLICATION

### Acidic nuclear phosphoprotein 32 (ANP32)

Multiple studies have indicated a key role for genes of the acidic nuclear phosphoprotein 32 (ANP32) family of proteins in influenza A virus replication by promoting viral polymerase activity (92, 93). The mechanism was shown to involve an essential role of ANP32 proteins as an assembly point for the influenza A virus replicase (94). Importantly, different members of the ANP32 gene family are required for influenza A virus replication in different host species. For example, influenza replication in chicken cells was shown to require ANP32A (95–97), whereas in mouse cells, ANP32B is required (98). Other species may utilize multiple ANP32 family members (92, 99), including some with differential use (100). Differences in amino acid sequences of ANP32 family proteins, such as N129I-D130N, create important species barriers because they require influenza to mutate to achieve efficient replication in another host (96, 101).

Leveraging the key role of ANP32A in influenza replication in chickens, one study performed gene editing to test influenza resistance in homozygous ANP32A N129I-D130N birds (102). Influenza challenge was performed intranasally with either 10^3^ (low dose) or 10^6^ PFU (high dose) of H9N2-UDL virus at 2 weeks of age. At the lower dose, most directly inoculated birds were protected from having detectable viral oropharyngeal shedding, and no transmission to contact birds was detected (102). However, for the high-dose experiment, five out of 10 inoculated N129I-D130N birds developed detectable shedding as well as a wild-type sentinel exposed to the edited birds. In that experiment, only sentinel birds with the homozygous N129I-D130N genotype remained undetectable for viral shedding (102). Thus, ANP32A N129I-D130N homozygous chickens displayed notable partial resistance to influenza. The high-dose experiment also led to compensatory viral mutations where mutants were able to overcome this resistance. The PA-349K/PB2-M631L double mutant virus was able to replicate in chicken embryos lacking ANP32A, and only chicken cells lacking ANP32A, ANP32B, and ANP32E did not support viral replication. The rapid development of viral escape is concerning for the application of ANP32A N129I-D130N alone to combat influenza virus, and the authors concluded that strong resistance based on an ANP32 mechanism would need to consider all three ANP32 genes (102).

## ADAPTIVE IMMUNE RESPONSE

### Major histocompatibility complex

Classical major histocompatibility complex (MHC) genes encode molecules that present peptides for T-cell immune inspection and play a key role in developing downstream adaptive immune responses. Influenza A viruses downregulate MHC class I and II gene expression in chicken cells to limit immune response (103, 104). Within live birds, one study examined the differences in MHC class I and class II gene haplotypes on dead and surviving chickens in Thailand in 2004 during their H5N1 HPAI outbreak. For the MHC class I genes, positive correlates of survival were seen with the MHC B21 homozygous haplotype, and a negative correlation (100% mortality) was seen with the MHC B13 haplotype (105). To directly test the resistance of MHC B21 and susceptibility of MHC B13 chickens, different congenic chicken lines were evaluated with a low-dose challenge of H5N1 virus. In two challenge trials, neither chicken line was resistant to infection, but differences in mortality rates were observed, with the MHC B21 chicken lines having lower mortality rates than the MHC B13 chicken lines. The data are supportive of the role of MHC haplotypes in resilience to HPAI challenges (106).

Additional studies have compared different chicken lines for relative resistance with both LPAI and HPAI viruses. One study compared two different congenic chicken lines, haplotype MHC B21 and B12, with several LPAI challenge viruses. The B21 haplotype birds for two viruses compared with the B12 birds appeared to have higher resistance to infection with lower cloacal shedding and reduced transmission to contact controls. However, when comparing MHC B21 and B12 genetic lines challenged with a highly chicken-adapted H9N2 virus, no genetic line differences in resilience were observed, indicating that resilience is viral strain-dependent (107). In an additional study using congenic MHC B21 and B12 lines, investigators reported differences in T cell numbers among the lines, and *in vitro* inoculation with a LPAI H7N2 virus of lung macrophages showed differential expression of immune genes between the lines. The B21 birds had a higher expression of anti-viral interferon genes, and the B12 birds had a higher expression of negative regulator genes. The conclusion was that the difference in clinical disease may be related to immunopathology (108). Some disease-related breeding strategies avoid directional selection on the MHC-associated alleles, attempting to maximize the range of potential pathogen recognition capacity and knowing that alleles beneficial for AIV can be detrimental to other pathogens (109).

## ADDITIONAL GENES/PATHWAYS

Genome-wide knockout screens have been performed with different strains of H5N1 in the human lung cell line, A549, to uncover novel host factors involved in AIV infection (16–19, 110). One screen was also performed with a chicken cell line to interrogate the genome for avian-specific host factors (15). A total of 706 genes were identified in the screen, with 599 novel genes not identified in previous screens in other species and considered avian-specific host factors. As previously described, *SLC35A1* was a top gene candidate in this screen, and the knockout DF-1 cells showed improved survivability after AIV infection. Other gene candidates from the screen that demonstrated improved survivability after knockout included *ZBTB6*, *PARP14*, *XKR7*, *CREB3L3*, *MOB1B*, *FRMD4A*, *NHE1*, *EPHX1*, *DCP1A*, *FASLG*, *EIF4G2*, *BBS5*, *ALS2CL*, *MRVI1*, *BMP7*, *RNF2*, and *AP2B1* (15). Additional investigation and refinement of these host factors will determine if some of the genes are specifically involved in AIV infection in poultry; however, other considerations, such as bird viability after knockout or detrimental effects on production efficiency, need to be assessed.

## FIELD AND EXPERIMENTAL MODEL COMPARISON WITH POTENTIALLY RESISTANT CHICKENS

Several studies have been published that examined surviving birds, mostly in layer breeds, after a HPAI outbreak to screen for genetic markers associated with survival. The presumption from these studies is that the surviving birds had been infected and that their unique genomic composition allowed them to survive. However, several additional factors can affect the clinical outcome of HPAI virus, including the strain of the virus used for the challenge. Virulence of strains within the HPAI category can vary significantly with certain strains, resulting in less than 100% mortality under standard pathotyping assays (1). Viruses of the goose/Guangdong lineage have been observed to be more virulent compared with most other HPAI viruses, but some birds may survive for reasons other than host genetics. Additional reasons for survival include co-infections that stimulate a strong innate immune response. For example, challenging birds with a mesogenic Newcastle disease virus (NDV) followed by a challenge with the HPAI virus resulted in surviving birds because of the strong innate immune response elicited by NDV (111). Previous exposure to LPAI strains can also provide both humoral and cellular immunity that may be protective against subsequent exposure to HPAI viruses (112, 113). Finally, the amount of virus needed to infect chickens, the chicken infectious dose 50, varies by strain, and experimental infections have shown that successful transmission does not always occur even in direct contact with birds (114, 115). Published studies from surviving birds may provide important clues about host resistance genes, but the field studies did not provide direct evidence that the surviving birds were actually infected, possibly co-infected with other viruses, or previously exposed to LPAI viruses that could have contributed to survival.

Several studies evaluated survivors from different HPAI outbreaks, and various methods of genomic evaluation were used to compare survivors with uninfected layer chickens of a similar genetic background. The samples were from Mexico during the 2012 H7N3 outbreak and from the United States during the 2015 H5N2 outbreak. The first study used a single-nucleotide polymorphism (SNP) array that could identify 600,000 SNPs across the genome. This SNP array analysis could locate regions of the chicken genome associated with resistance, but not the exact genes associated with the resistance phenotype (116). It showed a distribution of involved genomic regions on multiple chromosomes, especially chromosome 1. Several follow-up studies using the same database determined that the genetic association with resistance was different with the two virus strains evaluated, and with no common genetic resistance genes, it would be harder to breed chickens resistant to all AIVs (117). Several chromosomes were associated with resistance, and these chromosomes included regions with immunologic genes (118). A later study, using the same samples from the US 2015 outbreak, harnessed a full genome sequencing approach that further supported several gene regions associated with increased resistance, and candidate genes within these regions had previous associations with influenza replication or possible antiviral activity (119). These studies support the idea that multiple genes contribute to disease resilience, but their findings do not provide strong evidence of specific genes related to resilience or complete resistance to infection.

A series of studies evaluated genetic resistance by comparing the indigenous Vietnamese Ri chicken, specifically bred to have a resistant or susceptible phenotype based on the presence of a resistant/susceptible *Mx* gene, with the “resistant” MHC congenic B21 or “susceptible” MHC B13 haplotypes. Host responses were compared after challenge with low-dose virulent H5N1 virus; however, in these studies, tracheal or lung samples were only taken on 1 or 3 days post-challenge, which was before severe clinical disease was observed. No virological studies were included, nor was information on whether the resistant phenotype would have complete or only partial protection from morbidity or mortality. One study, using a gene set enrichment analysis on tracheal samples 3 days post-challenge, identified 78 genes with increased expression in the resistant chickens, including cytokines, chemokines, interferons and ISGs, Jak-STAT signaling pathway genes, and MHC class I and II and their co-stimulatory molecules (120). A second study using the same model identified the upregulation of many identical genes in the trachea, as confirmed by quantitative real-time RT-PCR (qRT-PCR) (121). A third study using RNA sequencing with confirmation by qRT-PCR on lung samples 3 days post-challenge also showed increases in cytokines, chemokines, and ISGs (122). A fourth study evaluated long non-coding RNAs and differentially expressed (DE) transcripts and found that Ri-resistant birds had higher levels of immune transcripts than the susceptible birds (123). A fifth study evaluated micro-RNA (miRNA) differential expression and discovered 44 DE miRNAs at 3 days post-challenge in lung samples. These miRNAs were associated with different immune genes that likely would play a role in the response to AIV infection (124). The final study compared exosomes, membrane vesicles-containing proteins, lipids, DNA, mRNA, and miRNAs, from the blood of the resistant and susceptible chickens at 1 and 3 days post-challenge. Using small RNA sequencing and confirmation by qRT-PCR, differences were observed with up- and down-regulation of miRNAs. Similar to previous studies, the miRNAs were related to cytokines, chemokines, and immune-related signaling pathways (125). Based on the relative resistance of the Ri chickens, correlations to immune genes were identified, but it remains unclear how this information can be incorporated with modern poultry genetics to increase disease resilience.

## POTENTIAL PATHS TO ACHIEVE INFLUENZA-RESISTANT POULTRY

### Natural genetic variation

Good examples of the use of naturally occurring genetic variation to combat evolving pathogens come from discoveries in wheat. Many regions of the world have utilized selective breeding to achieve wheat pathogen resistance as a primary (sometimes sole) disease management strategy, and these cases span many decades. The experience has largely been that the use of single gene resistance in the context of rapidly evolving pathogens has lasted 3–5 years in the field before the resistance has been overcome (126). On the other hand, durable resistance solutions have been achieved, for example, with wheat leaf rust, via combinatory approaches based on multiple sources of even partial resistance (126). An intentional combination of partial resistance sources with respect to different underlying mechanisms has been helpful and suggests a potential path for selective breeding interventions to combat many pathogens, including AIV. Another key lesson is the continuing search for new and different sources of pathogen resistance to build and maintain an ongoing effective strategy against pathogens that evolve quickly (126).

Although principles can be derived from other host-pathogen systems, the specific genetics must work for AIV resistance in poultry. We have explored many potential mechanisms, and others are being discovered. Poultry genetic companies routinely incorporate traits into overall selection indices based on the economic trait value. A note of caution on selection for any trait, especially for pathogen resistance, is the issue of negatively correlated traits. What improves one trait might diminish other traits. This has been shown for many systems, whether based on (i) competitive use of energy for immunity, growth, or reproduction, (ii) immune response balance to different types of pathogens, or (iii) other specific tradeoffs implicit in specific mechanisms of genetic resistance. One example comes from chickens selected for high- or low-antibody responses to sheep red blood cells (127). In that case, there was a negative genetic correlation with body weight, such that improved antibody responses were correlated with decreased body weight. However, the genetic loci involved were distinct, suggesting that it would be possible to achieve both increased body weight and increased antibody responses. This would need to be done through careful selection and, in some cases, identification of relatively rare recombinant chromosomes to combine desirable alleles from different genes. This example and many others (128, 129) highlight the need to test correlated responses to selection for AIV resilience or partial resistance traits before implementing genetic selection for improved AIV resistance/resilience.

### Gene editing

Although selective breeding has been widely successful within the poultry industry, particular traits, such as disease resistance, cannot always be acquired through this strategy due to the lack of genetic variation available. With the use of gene-editing technologies, novel alleles can be introduced into poultry populations in only a few generations that may confer resistance to pathogens. The adaptation of the clustered regularly interspaced short palindromic repeats (CRISPR)/CRISPR-associated nuclease 9 (Cas9) system from bacteria and archaea for use in animal systems has revolutionized the field of gene editing in livestock and poultry by providing an efficient and user-friendly platform to introduce these changes to the existing genetic sequence (130). As an illustration of the immense utility of the CRISPR/Cas9 system, PRRS virus-resistant pigs were created by knocking out the cluster of differentiation 163 (*CD163*) gene, which encodes the viral entry receptor (131). The *CD163* knockout pigs showed no evidence of viremia or antibody response when challenged with the PRRS virus, whereas parallel efforts to discover a naturally occurring PRRS virus-resistant pig by screening thousands of pigs from different genetic backgrounds proved unsuccessful (132). Although these genetic “silver bullets” exist, no target genes or loci have been identified in chickens to date that will completely prevent AIV infection. Impeding other mechanisms of the viral life cycle, including endocytosis, trafficking, and replication, may improve resilience, but the virus has entered the cell and likely will mutate to escape the edits, thus permitting infection.

As mentioned above, ANP32A has been identified as a key host factor for influenza virus replication, and gene editing was employed to induce a two-amino-acid change (N129I and D130N) in chicken ANP32A that prevented dimerization and interactions with the viral polymerase (102). This was the first application of CRISPR/Cas9-mediated gene editing for combating influenza virus in chickens and demonstrated that a combination of gene edits may be required for resistance. Another major revelation from this study was that AIV mutates rapidly to continue to replicate in its host; therefore, the virus will need to be monitored as additional gene edits are performed.

Moreover, targeting host genes needs to be performed with caution as deleterious host effects of the edit can occur, and changes need to be extensively characterized for growth and performance traits to ensure that production is not compromised. Additionally, edits to certain genes, such as *SLC35A1*, may result in embryonic lethality or severely compromise the homeostasis of the host (20, 21). Therefore, gene targets need to be thoroughly investigated prior to and after editing in the chicken.

## TRANSGENESIS

Another potential method of providing resistance to AIV is through transgenesis, which involves the insertion of a novel genetic sequence from a different species into the host genome. Targeted insertion of transgenes can be achieved with the CRISPR/Cas9 system or with homologous recombination. Previous transgenic approaches have been promising for creating an AIV-resistant chicken. For instance, transgenic chickens were generated that expressed a short-hairpin RNA that acts as a decoy to inhibit the viral polymerase and prevent replication (133). After inoculation with H5N1 HPAI virus (A/turkey/Turkey/1/2005) at 10^5^ egg infectious dose 50 (EID_50_), the transgenic birds became infected, but importantly, they did not shed and transmit the virus to sentinel non-transgenic and transgenic birds. Only one integration of the transgene was assessed, and expression of the short-hairpin RNA was unable to be determined; therefore, it is unknown whether other integrations may result in increased expression and resilience. Additional transgenic chickens have been produced that overexpressed various ISGs, including *IFIT5*, *IFITM1*, and *IFITM3* (134–136). These models demonstrated protection against the HPAI H5N1 virus at a low challenge dose (10^4^ EID_50_), but they succumbed to infection at a slightly higher dose (10^5^ EID_50_). Interpretation of these models is unclear, as mosaic birds were used for the challenge studies that do not have a homogeneous expression of the transgene in all cells of the body.

Additional genetic strategies to protect against AIV include the transgenic expression of CRISPR systems to restrict the virus. The CRISPR/Cas13 system, which targets RNA, has been inserted into the genome of DF-1 cells as a strategy to inhibit AIV (137). Four CRISPR RNAs (crRNAs) were expressed in the Cas13a DF-1 cells to target the RNA genome of the virus, specifically the PB1, NP, and M genes, and decreased viral titers were observed after infection (137). Although the expression of Cas13 appears to be a promising strategy to make transgenic birds with resilience to AIV, mouse models expressing Cas13 have demonstrated off-target, deleterious effects in the host associated with the transgene that may confound its utility (138).

With only a few studies to date, the use of gene editing or transgenesis to combat avian influenza virus infection has not been extensively investigated at the *in vivo* level in chickens. Nevertheless, a “silver bullet” or one gene that provides resistance to AIV infection is doubtful. Viable strategies for implementing gene editing to provide AIV resistance may include stacking multiple gene edits in a bird or combining gene edits with transgenes. Targeting multiple points in the viral infection life cycle, such as attachment, trafficking, and replication, appears to be the most promising strategy for robust resistance and avoiding breakthrough infections. Importantly, any gene edit or transgene must be fully characterized in the bird to confirm virus resilience or resistance as well as maintain the original level of performance.

## CONCLUSIONS

Many groups using multiple different approaches have tried to identify genetically resistant poultry with no success. Several genes have been identified that may increase genetic resilience, but because of the high virulence and genetic variability of HPAI viruses, no genes on their own are likely to provide enduring resilience to disease. However, other genes involved in AIV infection in chickens are likely to be identified and validated. An even greater obstacle is to introduce these genetic changes into commercial lines, where genetic trade-offs for disease resistance will need to be weighed against many other production traits. Future work will clarify these tradeoffs in the form of the potential correlated responses to selection for naturally occurring, gene-edited, or transgenic alleles. We note that gene editing and/or transgenic approaches can be used alone, in combination with naturally occurring variants and breeding, or as the first discovery step to implement through similar natural genetic variants and selective breeding. If successful, any of these approaches would provide additional tools to combat AIV and be immensely valuable to the poultry industry.
